# The repeatability of computed tomography lung volume measurements: Comparisons in healthy subjects, patients with obstructive lung disease, and patients with restrictive lung disease

**DOI:** 10.1371/journal.pone.0182849

**Published:** 2017-08-10

**Authors:** Jae Min Shin, Tae Hoon Kim, Seokjin Haam, Kyunghwa Han, Min Kwang Byun, Yoon Soo Chang, Hyung Jung Kim, Chul Hwan Park

**Affiliations:** 1 Department of Radiology and the Research Institute of Radiological Science, Gangnam Severance Hospital, Yonsei University College of Medicine, Seoul, Republic of Korea; 2 Department of Thoracic and Cardiovascular Surgery, Ajou University School of Medicine, Suwon, Republic of Korea; 3 Department of Radiology, Research Institute of Radiological Science, Severance Hospital, Yonsei University College of Medicine, Seoul, Republic of Korea; 4 Division of Pulmonology, Department of Internal Medicine, Gangnam Severance Hospital, Yonsei University College of Medicine, Seoul, Republic of Korea; New York University School of Medicine, UNITED STATES

## Abstract

In this study, we examined the repeatability of computed tomography (CT) lung volume measurements in healthy individuals and patients with obstructive and restrictive lung diseases. To do this, we retrospectively enrolled 200 healthy individuals (group 1), 100 patients with obstructive lung disease (group 2), and 100 patients with restrictive lung disease (group 3) who underwent two consecutive chest CT scans within a 1-year period. The CT lung volume was measured using a threshold-based, three-dimensional auto-segmentation technique at a default range from –200 to –1024 HU. The within-subject standard deviation, repeatability coefficient, within-subject coefficient variability, and intraclass correlation coefficient were evaluated. No significant differences were identified between the two consecutive CT lung volume measurements in any of the groups (*p*> 0.05). The within-subject standard deviations for groups 1, 2, and 3 were 441.1, 387.0, and 288.6, respectively, while the repeatability coefficients were 1222.6, 1072.6, and 800.1, respectively. The within-subject coefficient variabilities for groups 1, 2, and 3 were 0.097, 0.083, and 0.090, respectively, while the intraclass correlation coefficients were 0.818, 0.881, and 0.910, respectively. The two CT lung volume measurements showed excellent agreement in healthy individuals and patients with obstructive or restrictive lung disease. However, the repeatability was lower in healthy individuals than it was in patients with lung diseases.

## Introduction

Measurements of lung volume are useful for diagnosing and stratifying lung diseases. Moreover, the lung volume can help predict postoperative lung function in patients with chronic lung diseases who are considering lung transplantation or lung volume reduction surgery [1–4]. Traditionally, the functional lung volume, which is measured with a pulmonary function test (PFT), is used for these purposes [5–7].

Recently, however, use of the computed tomography lung volume (CTvol) has been increasing and playing an essential role in respiratory medicine. With advances in quantitative CT analysis techniques, precise lung volumes can now be measured [8]. The CTvol has been used to objectively measure the severity of lung diseases, which can in turn help clinicians plan suitable interventions for those patients displaying severe lung disease [1–3,9]. Additionally, the CTvol has become an important tool for developing new disease-specific therapies and for identifying candidates who would benefit the most from these new treatments [1–4,10–13]. The major limitation of CTvol measurements is that the volume of the scanned lung changes according to the individual’s degree of inspiration during the CT scan.

Establishing repeatability is therefore necessary in order for CTvol measurements to be used as standard parameters in clinical settings [1–3]. Although several studies on the repeatability of CTvol exist, they mostly utilized patients with chronic obstructive pulmonary disease (COPD) [14]; hence, little is known about the repeatability of CTvol in healthy individuals and patients with lung diseases other than COPD. The purpose of the present study was to evaluate the repeatability of CTvol by comparing two repeated CTvol measurements in healthy individuals, patients with obstructive lung disease, and patients with restrictive lung disease.

## Materials and methods

This retrospective, observational study was approved by the institutional review board at our institution (Gangnam Severance Hospital). Since all CT images and clinical data were retrospectively obtained from medical records, the requirement for informed consent was waived.

The sample sizes required to perform a statistical analysis of the repeatability of CTvol measurements in three groups (~200 healthy participants, ~100 patients with obstructive lung disease, and ~100 patients with restrictive lung disease) were determined using methods described below (see *Statistical Analysis*).

We retrospectively reviewed the medical records of 207 healthy participants with normal PFT results (forced expiratory volume 1/forced vital capacity [FEV_1_/FVC] ratio ≥70% and FVC [%] ≥80%) [15,16] who underwent two consecutive low-dose chest CT scans within 1 year for the purpose of lung cancer screening via self-referral from January 2013 to December 2015. Patients with atelectasis (n = 5) or active infectious lung conditions (n = 2) were excluded. Additionally, the records of 123 patients with obstructive lung disease (FEV_1_/FVC ratio <70% on PFT) and 109 patients with restrictive lung disease (FEV_1_/FVC ratio ≥70% and FVC [%] <80% on PFT) who underwent two consecutive chest CT scans and PFTs within 1 year were reviewed consecutively. Among the 123 patients with obstructive lung disease, patients with incomplete medical records (n = 5), tumorous conditions (n = 4), atelectasis (n = 9), active infectious lung conditions (n = 3), or pleural effusion (n = 2) were excluded. Among the 109 patients with restrictive lung disease, patients with incomplete medical records (n = 2), tumorous conditions (n = 1), atelectasis (n = 1), active infectious lung conditions (n = 2), or pleural effusion (n = 3) were excluded. Finally, 200 healthy individuals (group 1), 100 patients with obstructive lung disease (group 2), and 100 patients with restrictive lung disease (group 3) were enrolled.

### CT protocol

One of the following four scanners was used for the chest CT scans: Somatom Sensation 16, Somatom Sensation 64, Somatom Definition AS+ (all Siemens Medical Solutions, Erlangen, Germany), or Brilliance 64 CT (Philips Healthcare, Best, Netherlands). Participants were scanned in the supine position from the lung apex to the adrenal glands during breath-holding at the end of inspiration. Guidance was provided to the participants during the scan through the simple audio-recorded instruction, “inhale and hold your breath.” Chest CT was performed using a helical technique and a mediastinal window setting with the following exposure parameters: 120 kVp, 50–130 mA, and slice thickness = 1–3 mm. The data were reconstructed with 1–3 mm intervals on the scanner workstation. All CT images were sent to the picture archiving and communication system (Centricity 2.0; GE Medical Systems, Mount Prospect, IL, USA) for analysis.

### CT image analysis

Two radiologists (THK and CHP, with 22 and 10 years of experience in chest radiology, respectively) analyzed all of the CT scans. Images were excluded if abnormal features such as previous thoracic surgery, tumors, active lung infectious conditions, atelectasis, or large amounts of pleural effusion were found. The three-dimensional volume measurements were performed on axial CT images using a commercially available reconstruction program (Aquarius iNtuition^™^ Ver.4.4.6; TeraRecon, Foster City, CA, USA). A threshold-based, three-dimensional auto-segmentation technique was used to semi-automatically measure the CTvol, which was used as the reference anatomical lung volume, at a default range from –200 to –1024 HU.

### Statistical analysis

Sample size calculation was performed using 30 individuals who were not included in the final study population and was based on the minimum amount of change in the lung volume considered to indicate a clinically meaningful difference to a patient (δ) [17]. We found that at least 192 participants were required to have the upper limit of the 95% confidence interval of the repeatability coefficient be ≤90% of δ with two replications. Moreover, we found that at least 98 participants were required to have the upper limit of the 95% confidence interval of the repeatability coefficient be ≤85% of δ with two replications.

Continuous variables are summarized as the mean ± the standard deviation (SD), and categorical variables are summarized as frequencies or percentages. Normality assumptions for continuous variables were confirmed with the Shapiro-Wilk test. Analyses of variance with Bonferroni post-hoc tests were used to evaluate the statistical differences in the demographic and PFT values among the three groups. Paired *t*-tests were used to evaluate the differences between the two consecutive CTvol measurements, and Pearson’s correlation analyses were performed to compare the two consecutive CTvol values in the three groups, respectively. Bland-Altman analyses were used to determine the limits of agreement between the two consecutive CTvol values in the three groups [18]. To assess the repeatability of the CTvol measurements in each participant, repeatability statistical metrics were calculated in the three groups using a random-effects model that included a random intercept for each participant. The metrics included the following: (1) within-subject standard deviation (wSD), defined as the estimated SD of the repeated measurements from a single participant, measured over replicates; (2) repeatability coefficient (RC), defined as the least significant difference between two repeated measurements taken under identical conditions at a two-sided significance of α = 0.05; (3) within-subject coefficient of variation (CV), defined as the relative variability (the SD divided by the mean); and (4) intraclass correlation coefficient (ICC), defined as the proportion of total error that was not associated with measurement error [19].

The generalized estimating equation approach with an identity link function and independent working correlation structure were used to evaluate the statistical differences in the wSD, RC, and CV among the three groups. Inter-observer reproducibility in measuring the CTvol was evaluated with the ICC. ICCs of <0.40, 0.40–0.75, and 0.76–1.00 signified poor agreement, fair to good (moderate) agreement, and excellent agreement, respectively. Differences were considered significant if the *p*-value or Bonferroni-adjusted *p*-value was less than 0.05.

All statistical analyses were performed using commercially available software, i.e., SPSS Statistics for Windows version 20.0 (IBM Corp., Armonk, NY, USA) or SAS version 9.4 (SAS Institute Inc., Cary, NC, USA).

## Results

### Participant demographics

The demographic data of the 200 healthy participants (group 1, males:females = 129:71, mean age: 53.6 ± 9.1 years), 100 patients with obstructive lung disease (group 2, males:females = 79:21, mean age: 68.0 ± 10.7 years), and 100 patients with restrictive lung disease (group 3, males:females = 52:48, mean age: 64.2 ± 12.8 years) are summarized in Table 1. The mean FVC, FEV_1_, and FEV_1_/FVC were significantly different among the three groups (*p*<0.001). Compared to group 1, the PFT results in group 2 revealed a typical obstructive pattern, including significantly lower FEV_1_, and FEV_1_/FVC. In group 3, the PFT results were characterized by restrictive ventilation, with lower FVC compared togroup 1 and a normal FEV_1_/ FVC.

**Table 1:** Demographic data from the healthy participants (group1), patients with obstructive lung disease (group 2), and patients with restrictive lung disease (group 3).

	Group 1 (n = 200)	Group 2 (n = 100)	Group 3 (n = 100)	*p*-value
**Males:Females**	129:71	79:21	52:48	<0.001
**Age (years)**	53.6 ± 9.1	68.0 ± 10.7	64.2 ± 12.8	<0.001*
**Height (cm)**	166.7 ± 8.2	165.5 ± 7.8	161.5 ± 8.7	<0.001^†^
**Weight (kg)**	67.1 ± 12.5	62.9 ± 12.5	61.9 ± 11.8	0.001^‡^
**FVC (%)**	97.2 ± 9.9	85.3 ± 17.9	67.9 ± 14.5	<0.001*
**FEV_1_(%)**	104.6 ± 11.8	67.1 ± 21.2	76.3 ± 16.3	<0.001*
**FEV_1_/FVC**	79.4 ± 5.7	53.3 ± 13.5	80.1 ± 9.2	<0.001*

Data are presented as the mean±the standard deviation, unless otherwise stated

*FVC* forced vital capacity, *FEV*_*1*_ forced expiratory volume in 1 s

*Group 1 vs. Group 2 <0.05, Group 2 vs. Group 3 < 0.05, and Group 3 vs Group 1 < 0.05

^†^ Group 2 vs. Group 3<0.05

^‡^ Group 1 vs. Group 2<0.05

### CTvol measurements

CTvol measurements were successfully obtained in all participants. The first and follow-up lung volumes in each group are listed in Table 2. No significant differences were noted between the two consecutive CTvol measurements in groups 1, 2, or 3 (*p* = 0.751, 0.744, and 0.371, respectively; Table 2).

**Table 2:** Two consecutive computed tomography lung volume measurements in healthy participants (group1), patients with obstructive lung disease (group 2), and patients with restrictive lung disease (group 3).

	Volume 1 (mL)	Volume 2 (mL)	*p*-value	Scan interval (days)
**Group 1**	4525.8 ± 1056.4	4539.9 ± 1009.6	0.751	361 (293, 365)
**Group 2**	4657.6 ± 1138.4	4639.6 ± 1102.8	0.744	279 (30, 365)
**Group 3**	3234.7 ± 947.1	3198.0 ± 978.6	0.371	182 (24, 365)

Data are presented as the mean±the standard deviation, unless otherwise stated.

The median interval between the two CT scans is presented with the minimum and maximum values.

**Fig 1:**
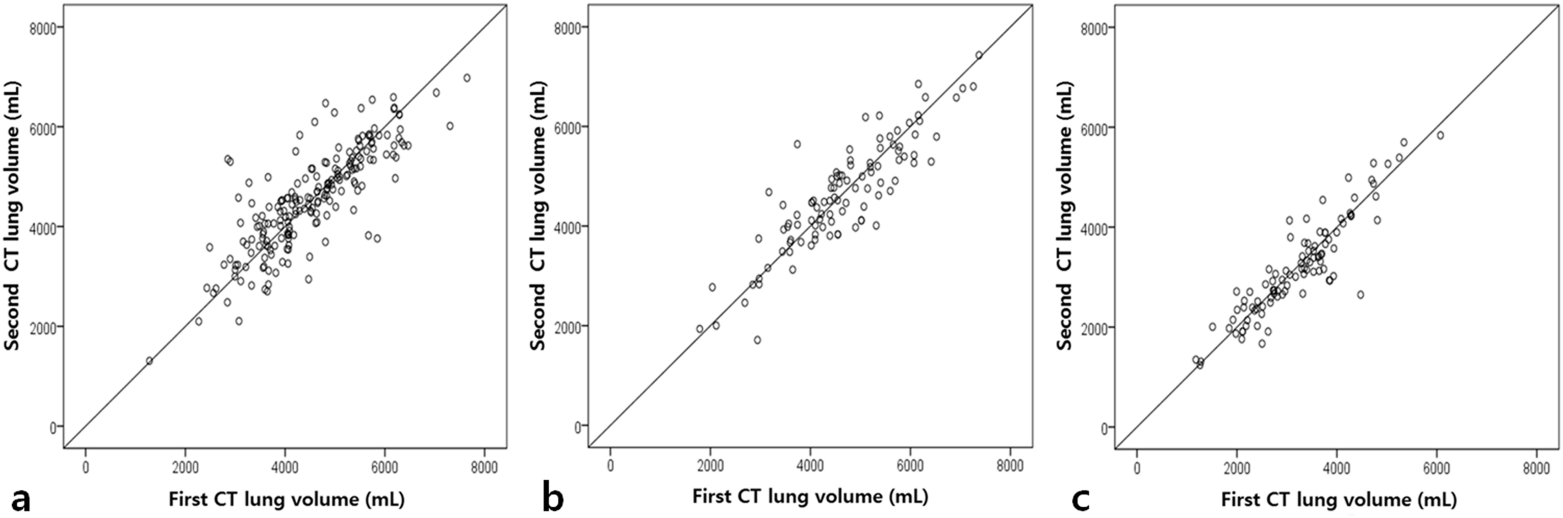
Comparisons between the two consecutive computed tomography (CT) lung volumes in healthy participants (group 1), patients with obstructive lung disease (group 2), and patients with restrictive lung disease (group 3). Plots show the lung volumes from the first and follow-up CT scans. From left to right are the correlations from the two CT scans in **(a)** group 1 (n = 200, correlation coefficient (r) = 0.818, p<0.01), **(b)** group 2 (n = 100, r = 0.880, p<0.01), and **(c)** group 3 (n = 100, r = 0.910, p<0.01).

The correlation coefficients between the two consecutive CTvol measurements were 0.818 in group 1, 0.880 in group 2 and 0.910 in group 3 (p<0.01) (Fig 1).

**Fig 2:**
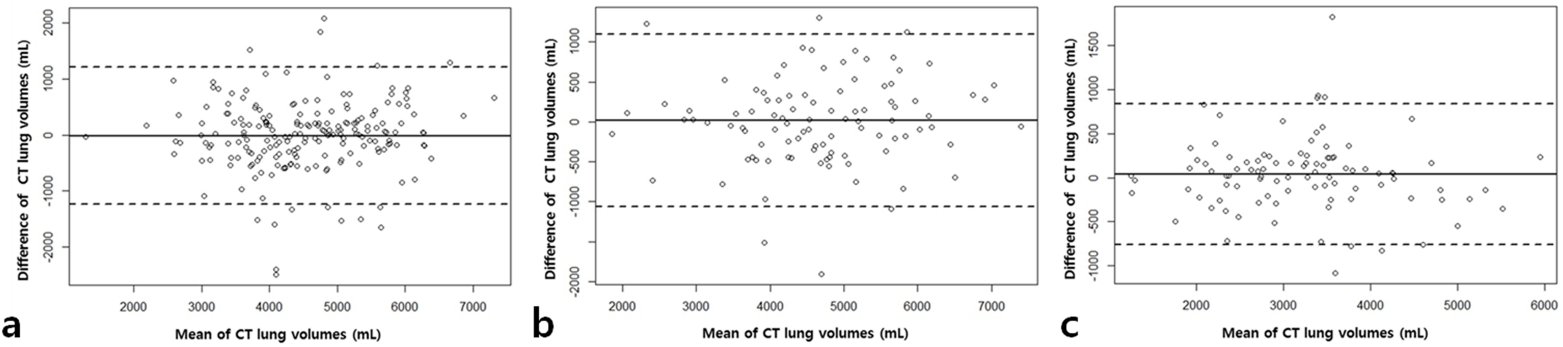
Bland-Altman plots of the correlations between the two consecutive computed tomography (CT) lung volumes in healthy participants (group1), patients with obstructive lung disease (group 2), and patients with restrictive lung disease (group 3). **(a)** in group 1, **(b)** group 2,**(c)** group 3. The dashed line indicates the mean bias, while the solid line indicates ±1.96 standard deviation.

The mean differences between the two consecutive CTvol measurements were-14.1 mL (95% limit of agreement: -1239.4 mL and 1211.3 mL) in group 1, -64.4 mL (95% limit of agreement: -1059.4 mL and 1095.4 mL) in group 2, and 36.7 mL (95% limit of agreement: -764.1 mL and 837.5 mL) in group 3 (Fig 2).

### Repeatability of CTvol measurements

The wSDs for the two consecutive CTvol measurements in groups 1, 2, and 3 were 441.1, 387.0, and 288.6, respectively. The RCs of groups 1, 2, and 3 were 1222.6, 1072.6, and 800.1, respectively. The CVs for groups 1, 2, and 3 were 0.097, 0.083, and 0.090, while the ICCs were 0.818, 0.881, and 0.910, respectively (Table 3). No statistical differences in the wSD and RC were identified between groups 1 and 2 (*p* = 0.613). However, group 3 had significantly smaller wSD and RC values than did groups 1 and2 (*p*<0.001). The CV was not significantly different among the three groups (*p*>0.05). The ICC for the inter-observer reproducibility in measuring the CTvol was very high (0.999, *p*< 0.001).

**Table 3:** Repeatability of the CTvol measurements in healthy participants (group1), patients with obstructive lung disease (group 2), and patients with restrictive lung disease (group 3).

Group	Mean (mL)	wSD (95% CI)	RC (95% CI)	CV (95% CI)	ICC (95% CI)
**Group 1**	4532.9	441.1 (401.8–489.0)	1222.6 (1112.9–1354.5)	0.097 (0.089–0.108)	0.818 (0.766–0.859)
**Group 2**	4648.6	387.0 (340.0–449.2)	1072.6 (941.7–1244.1)	0.083 (0.073–0.097)	0.881 (0.828–0.918)
**Group 3**	3216.4	288.6 (253.6–335.0)	800.1 (702.4–928.0)	0.090 (0.079–0.104)	0.910 (0.870–0.939)

*wSD* within-subject standard deviation, *CI* confidence interval, *RC* repeatability coefficient, *CV* within-subject coefficient variability, *ICC* intraclass correlation coefficient

## Discussion

In the present study, our CTvol measurements were reliable, demonstrating clinically acceptable RCs, similar CVs, and relatively high ICCs in all three groups. However, the ICC in healthy participants (0.818) was smaller than the ICCs in the two patient groups (0.881 in patients with obstructive lung disease and 0.910 in patients with restrictive lung disease). The estimated relative variabilities in the CTvol between the two CT scans from the same participant were <10% of the mean CTvol (CV = 0.097, 0.083, and 0.090 in groups 1, 2, and 3, respectively).

As mentioned above, the repeatability of CTvol measurements has mainly been studied in patients with COPD. For instance, Brown et al. [14] found that CTvol measurements acquired within a 9-month interval in 246 patients with COPD were highly repeatable (at total lung capacity: n = 126, *r* = 0.94, ICC = 0.943; at residual volume: n = 120, *r* = 0.89, ICC = 0.814) in their multicenter trials with precisebreath-hold coaching of the participants. Coxson et al. [1] also reported that quantitative CT measurements of the lung volume acquired during routine inspiratory chest CT scans in patients with emphysema were highly repeatable (n = 29, ICC = 0.99, mean difference with 95% confidence interval = 0.04 ± 0.08 L). Here, using the simple audio-recorded instructions “inhale and hold your breath,” the two consecutive CTvol measurements obtained within a 1-year interval from 100 patients with obstructive lung disease were highly repeatable (ICC = 0.881). The CTvol measurements were the most repeatable (n = 100, ICC = 0.910) in patients with restrictive lung disease compared to healthy individuals and patients with obstructive lung disease. The high repeatability of the CTvol measurements in patients with restrictive lung disease may be attributed to reduced lung compliance from stiff lung parenchyma, wherein more inspiratory effort is required to inflate the alveoli, resulting in less-variable inspiratory volumes [20].

Compared with the ICC in the study by Brown et al. [14], the ICC for patients with obstructive lung disease in our study was smaller, demonstrating less repeatability. The reduced repeatability of the CTvol measurements in our study likely resulted from the retrospective nature of the study and the use of simple audio-recorded instructions during the CT scans. In contrast, the study by Brown et al. [14] utilized clear breathing instructions and coached the participants on how to reach total lung capacity or residual volume during the CT scans. With better and more-consistent breathing instructions, the repeatability of CTvol measurements is expected to improve, especially in healthy individuals, who showed the lowest repeatability among the three groups in our study. Healthy individuals might have preserved lung compliance and elastance, with more variable or flexible degrees of inhalation, which may have resulted in the smaller ICC we observed in this group compared to the other two patient groups [20]. Our study showed that the CTvol measurements in healthy participants were as reliable but less repeatable than were the CTvol measurements in patients with lung diseases.

The current study has some limitations. First, this was a retrospective, single-center study. Hence, the sample sizes were too small to be able to generalize the repeatability of the CTvol measurements to other populations and patient groups. Second, the CT scans were separated by a relatively long time interval, which may have resulted in significant changes in lung volume. However, we confirmed that the lung volume changes in the three groups did not tend to increase or decrease. Third, all of the CT scans were routinely performed using simple audio-recorded instructions from the scanner. To obtain reliable CT images, more unified scanning techniques should be used, along with proper training to ensure consistent inspiration during the chest CT scans. Therefore, prospective, multicenter studies with careful training regarding how to breathe during the CT scan are needed to verify the reliability and repeatability of CTvol measurements.

In conclusion, the ICC for the repeated CTvol measurements was the smallest in healthy participants. Despite this, CTvol measurements obtained using routine inspiratory chest CT scans appear to be highly repeatable in healthy individuals and patients with obstructive or restrictive lung diseases. The estimated relative variabilities of the two consecutive CTvol measurements were less than 10% in all three groups.

## Supporting information

S1 File Attached files are data of two consecutive computed tomography lung volume measurements in the healthy participants (group1), patients with obstructive lung disease (group 2), and patients with restrictive lung disease (group 3). (XLSX)
